# A comprehensive review of summer savory (*Satureja hortensis* L.): promising ingredient for production of functional foods

**DOI:** 10.3389/fphar.2023.1198970

**Published:** 2023-07-24

**Authors:** Afaf Ejaz, Sadaf Waliat, Muhammad Sajid Arshad, Waseem Khalid, Muhammad Zubair Khalid, Hafiz Ansar Rasul Suleria, Marian-Ilie Luca, Costel Mironeasa, Ana Batariuc, Mădălina Ungureanu-Iuga, Ionica Coţovanu, Silvia Mironeasa

**Affiliations:** ^1^ Department of Food Science, Faculty of Life Sciences, Government College University, Faisalabad, Pakistan; ^2^ University Institute of Food Science and Technology, The University of Lahore, Lahore, Pakistan; ^3^ Department of Agriculture and Food Systems, The University of Melbourne, Melbourne, VIC, Australia; ^4^ Faculty of Food Engineering, “Ştefan cel Mare” University of Suceava, Suceava, Romania; ^5^ Faculty of Mechanical Engineering, Automotive and Robotics, “Ştefan cel Mare” University of Suceava, Suceava, Romania; ^6^ Integrated Center for Research, Development and Innovation in Advanced Materials, Nanotechnologies, and Distributed Systems for Fabrication and Control (MANSiD), “Ştefan cel Mare” University of Suceava, Suceava, Romania

**Keywords:** antimicrobial activity, chemical composition, antioxidant activity, health benefits, nutraceutical, additive, health benefits γ-terpinene, food applications

## Abstract

This review aims to measure the different aspects of summer savory including biological activity, medicinal properties, nutritional value, food application, prospective health benefits, and its use as an additive in broiler feed. Furthermore, toxicity related to this is also overviewed. Summer savory leaves are abundant in total phenolic compounds (rosmarinic acid and flavonoids) that have a powerful antioxidant impact. Rosmarinic (α-O-caffeoyl-3,4-dihydroxy-phenyl lactic) acid has been identified in summer savory as a main component. According to phytochemical investigations, tannins, volatile oils, sterols, acids, gums, pyrocatechol, phenolic compounds, mucilage, and pyrocatechol are the primary compounds of *Satureja* species. Summer savory extract shows considerable biological potential in antioxidant, cytotoxic, and antibacterial assays. Regarding antioxidant activity, summer savory extract displays an inhibitory effect on lipid peroxidation. Summer savory also has Fe (III) reductive and free radical scavenging properties and contains minerals and vitamins. Summer savory has important biological properties, including antimicrobial activity and antioxidant activity, and protective effects against Jurkat T Cells, Alzheimer’s disease, cancer, infection, cardiovascular diseases, diabetes, and cholesterol. The leaves and stems of this plant are employed in the food, feed, and pharmacological industries due to their antioxidant properties and substantial nutritional content. Conclusively, summer savory is widely considered beneficial for human health due to its versatile properties and medicinal use.

## 1 Introduction

Summer savory (*Satureja hortensis* L.) is one of the most popular varieties of savory. It is a seasonal herb that displays similar function and flavor to the perennial winter savory. Autumn savory is used more regularly due to its bitter taste. From July to September in the Northern Hemisphere, this herb blossoms with violet tubular flowers. It has relatively thin brass foliage and rises to a height of 30–60 cm (1–2 ft). *Satureja hortensis* L. is a renowned herb in eastern Canada, and it can be used similarly to sage (Burlando et al., 2010). It is the predominant ingredient in many condiments for fowl, and it is used to produce cretonnade (cretonade). Summer savory is a very rich chicken vinaigrette that can be served with turkey, goose, and duck. It is also used in other food products, including fricot and mince pies. It is frequently accessible in dried form throughout the year in grocery stores, and it is used in varying amounts; for example, it is used in large quantities in cretonnade, whereas it is consumed in smaller quantities in other food products (Brown, 2009). It is popular for seasoning grilled meats and for barbecues, stews, and sauces. Summer savory is recommended for use in sausages over winter savory due to its richer aroma. It is used frequently in Bulgarian dishes, imparting a powerful fragrance in a wide range of meals. The traditional food of Sofia contains three ingredients for seasoning instead of just salt and pepper: salt, crimson chili, and summer savory. Sharena sol is the result of combining these ingredients. In Romanian cuisine, summer savory, also known as “cimbru,” is used in “sarmale” (stuffed cabbage and grape leaf rolls) and “mititei” (grilled ground meat rolls) (Cutler et al., 2010). Savory can grow from seeds propagated in a moderately fertile environment. It takes a long time to germinate. Spring season plants are frequently trimmed in June for new usage. The plants can be picked and dried for winter usage when they are in flower (Nybe, 2007). Apart from food preparation, this herb has been used as a traditional antibacterial medicine for gastrointestinal issues (Gelovani et al., 2012). Georgia cultivates native hybrids of summer savory (Akhalkatsi et al., 2012). For instance, Kondari is a variety containing one of the largest total flavonoid concentrations along with the strongest hydro antioxidant activity levels, as discovered in our earlier research on Georgian spices (Rodov et al., 2010). In principle, phenylpropanoid is a precursor of rosmarinic acid, which is the plant kingdom’s second leading ester of caffeic acid. Animal investigations have reported that *S. hortensis* powder and its polyphenolic fraction display anti-inflammatory characteristics (Hajhashemi et al., 2002; Uslu et al., 2003). To some extent, this activity has been credited to rosmarinic acid, which has been shown to have anti-inflammatory and anti-allergic effects in human and animal studies (Sanbongi et al., 2003). The antiallergic action of rosmarinic acid has been related to two distinct mechanisms, namely, reactive oxygen species filtration and modification of the inflammatory process (Osakabe et al., 2004). For instance, the nephroprotective impact of rosmarinic acid has been related to an increased antioxidant potency, particularly higher glutathione levels and the antioxidant impact of enzymes (Tavafi and Ahmadvand, 2011).

Summer savory (*Satureja hortensis *L.) also contains a variety of volatile oils (carvacrol and thymol) that have anti-inflammatory (Can Baser, 2008; Hashemipour et al., 2014), antioxidant (Güllüce et al., 2003), antimicrobial (Şahin et al., 2003), and antifungal (Boyraz and Özcan, 2006) properties. Summer savory extract may be valuable to the poultry industry. A previous study reported that dietary summer savory essence (SSE) may enhance and maintain broiler chicken productivity efficiency, blood components, immunological reaction, and ileal microbiota (Movahhedkhah et al., 2019). Furthermore, regarding the volatile oil composition, carvacrol and γ-terpinene are the primary components identified in a typical essential oil of this herb. Therefore, this review comprises different elements relating to summer savory, including an update on the chemical composition of summer savory and its known biological and medicinal properties associated with active substances. Interestingly, specific findings regarding the toxicity of its herb extracts are also provided.

## 2 Chemical composition of summer savory

Many people in the food industry (herb, vegetable, and fruit growers) see improving human health as a principal goal for this century. Mineral elements are crucial for growth and can support and sustain the human ability to avoid ailments (Grusak, 2002). Herbs are valuable sources of quickly absorbable mineral elements among crops (Boyraz and Özcan, 2006). Summer savory (*Satureja hortensis* L.) is a common herbaceous plant from the Lamiaceae family that is grown in various regions across the world (Jafari et al., 2016) (Kudełka and Kosowska, 2008). Significant levels of minerals (potassium, phosphorus, calcium, magnesium, iron, and sodium) and vitamins (niacin, pyridoxine, riboflavin, thiamine, vitamin A, and vitamin C) were detected in the raw material of *S. hortensis* L. (Mumivand et al., 2010; Karimi et al., 2012; Soltani et al., 2014). Therefore, *S. hortensis* L. may be used as a nutritional basis for essential human minerals. The mineral rank of florae is critical not only for the nutritional content of food but also for the development, growth, and yield of crops. Crucial oils derived from several classes (Khan et al., 2020) in this family possess organic roles including physical function (photosynthesis) and environmental purpose (relationships between the flowers and their environment). Furthermore, the chemical structure of the oils from different *Satureja* strains has been discovered to vary greatly (Slavkovska et al., 2001). Various studies have shown that tannins, volatile oils, sterols, acids, gums, pyrocatechol, phenolic compounds, mucilage, and pyrocatechol are the main compounds of *Satureja* species.

**FIGURE 1:**
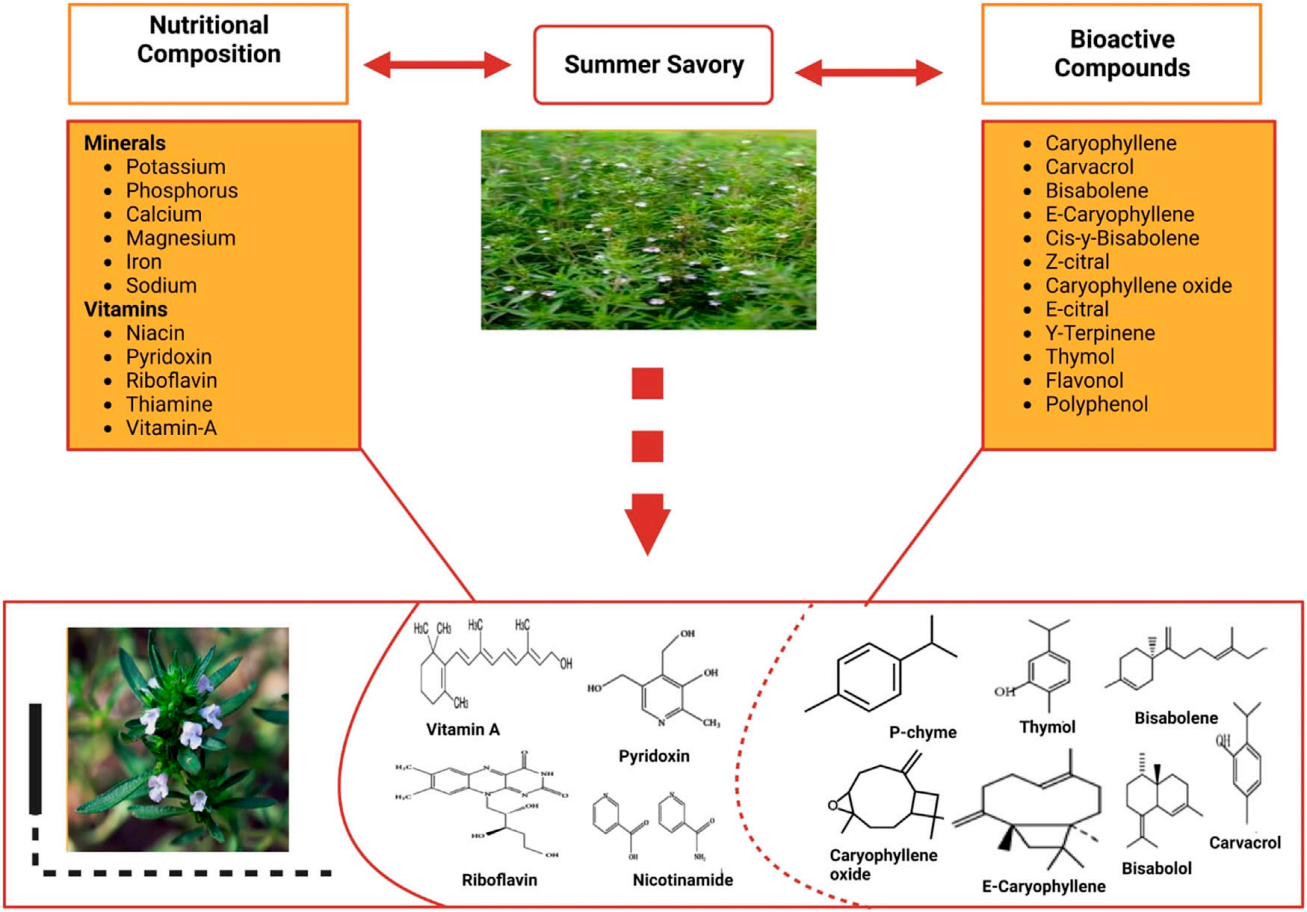
Chemical composition of summer savory.

Previous studies have shown the chemical composition of the oils from diverse *Satureja* species varies (Figure 1). The contents of the oils are determined by climatic, periodic, and terrestrial circumstances as well as yield period and purification practice (Baydar et al., 2004). Toward chemical profiling, water has been used to extract crucial oils from air-dried plants and strong spores through 4-h distillation in a Clevenger-type apparatus. Summer savory leaves and seeds had a total of 23 and 24 components, respectively (Farmanpour-Kalalagh et al., 2020). Furthermore, summer savory seeds contain chemicals including Carvacrol, Estragole (Methyl Chavicol), Caryophyllene, and E-Caryophyllene, whereas the leaves are a good source of Carvacrol, γ-Terpinene, and p-Cymene. Moreover, some chemical components are present in both (seeds and leaves), including Carvacrol, Caryophyllene, E-Caryophyllene, β-Bisabolene, cis-α-Bisabolene, Caryophyllene oxide, Z-Citral, E-Citral, γ-Terpinene, and δ-3-Carene (Farmanpour-Kalalagh et al., 2020). Dried summer savory is composed of volatile oil, which is a vital source of chemicals including carvacrol thymol and monoterpene hydrocarbons (beta-pinene, p-cymene, limonene, and camphene). Vitamins and minerals are present in the leaves of summer savory (Hamidpour et al., 2014). The results of many studies suggest that different parts of summer savory are chemically composed of Estragole, Carvacrol, E-Caryophyllene, Caryophyllene γ-Terpinene, Carvacrol, Thymol and p-Cymene, Caryophyllene, Carvacrol, β-Bisabolene, E-Caryophyllene, cis-α-Bisabolene, Z-Citral, Caryophyllene oxide, E-Citral, γ-Terpinene, and δ-3-Carene (Adiguzel et al., 2007; Kizil et al., 2009; Mahboubi and Kazempour, 2011; Katar et al., 2017; Mohtashami et al., 2018) (Figure 1).

## 3 Different extraction methods of active compounds from summer savory

The cytotoxic, antioxidant, and bactericidal properties of several *Satureja hortensis* L. extracts have been investigated to perform genetic characterization (Cvetanović et al., 2017). Different separate tests relying on a distinct mechanism have been used in order to evaluate the antioxidant activity of the extracts, including total antioxidant activity, lipid peroxidation inhibition, and hydroxyl radical scavenging (Cvetanović et al., 2017). Phenolic content, including condensed tannins, anthocyanins, and gallotannins, is determined in extracts that are associated with biological activities. Flavonoids are extracted by traditional extraction methods, while rutin is predominantly extracted using unconventional methods (Brighente et al., 2007; Fatima et al., 2023). The previously mentioned bioactive molecules can be separated in different ways (Table 1), i.e., via conventional methods (maceration, solvent extraction, soxhlet extraction, and vapor or hydrodistillation) (Waliat et al., 2023) and innovative technologies (emulsion liquid membrane, ultrasound-assisted extraction, enzyme-associated extraction, pulsed electric field, microwave-assisted extraction, and supercritical fluid) (Aadil et al., 2015; Maqbool et al., 2022; Arshad et al., 2023). A previous study was conducted on the extraction of the chemical composition and biological potential of summer savory extracts using conventional and nonconventional methods. The results verified the domination of the subcritical water approach for the isolation of natural compounds, followed by microwave-assisted extraction (Mašković et al., 2017). Another study was conducted on the extraction of essential oils from summer savory extract, whereby Two extraction methods were compared, namely, microwave-assisted hydrodistillation (MAHD) and traditional hydrodistillation (HD) methods. The outcomes confirmed that the novel method is more suitable compared to the traditional method (Rezvanpanah et al., 2011). The current study of Šeregelj et al. (2022) evaluates the biological activities of ultrasound- and microwave-assisted extracts of S. kitaibelii. The findings confirm that microwave-assisted extraction with water solvent is a promising approach (Šeregelj et al., 2022).

**TABLE 1:** Extraction of chemical compounds from Summer Savory.

Part of summer savory	Extraction method	Bioactive compounds	Solvent	Reference
Oil (Ariel part)	Conventional	Carvacrol, a-pinene, p-cymene, c-terpinene, and thymol methyl ether	Water/steam	Silva et al. (2005), Skočibušić et al. (2006)
Leaves, flower buds, and calyx	UV-visible spectrophotometry	Flavonoids	Ethanol	Khlebnikova et al. (2022)
Ariel part	Mass spectrometer	Rosmarinic acid, caffeic acid and naringenin acid	Methanol	Boroja et al. (2018)
Flowers, leaves, and steam	Conventional	Phenolic and flavonoids	Ethanol	Predescu et al. (2020)
Flowers	Non-conventional	Rutin and quercetin	Ethanol	Mašković et al. (2017)
Leaves	—	Carvacrol and γ-terpinene	—	Radácsi et al. (2016)
Seeds	Conventional	Carvacrol, c-terpinene, para-cymene; and the minor components a-terpinene, myrcene, camphene, and a-pinene	—	Svoboda and Greenaway (2003)
	GC and GCMS			
Blooming and shade-dried plants	HPTLC and HPLC	Rosmarinic acid (RA), caffeic acid (CA), chlorogenic acid (ChA), apigenin, luteolin, catechin, quercetin, rutin, and hyperoside	Methanol	Shanaida et al. (2021)
Dried	NMR	Luteolin, apigenin, and quercetin	Ethanol and acetone	Exarchou et al. (2002)
Oil (flowers)	GC-MC	α-Phellandrene and myrcene	Helium	Stankov et al. (2022)
Oil	GC-MC	Limonene	Helium	Stankov et al. (2022)
Extract (Ariel parts)	UHPLC/DAD/HESI-MS/MS	hydroxycinnamic acids, caffeic and isoferulic acids	Acetic acid with water	Boroja et al. (2018)
Ariel parts	UHPLC/DAD/HESI-MS/MS	Flavonol (quercetin), flavonol glycosides (isoquercitrin, astragalin, quercitrin), and coumarin derivatives (aesculin and aesculetin)	Acetic acid with water	Boroja et al. (2018)
Dried	HPLC-DAD	Protocatechuic acid	Water and formic acid	Mašković et al. (2017)
Dried	HPLC-DAD	p-Hydroxybenzoic acid	Water and formic acid	Mašković et al. (2017)

## 4 An overview of the biological activities of summer savory extracts

Different extracts showed considerable biological potential in antioxidant, cytotoxic, and antibacterial assays (Exarchou et al., 2002). Specifically, the extracts obtained by subcritical water extraction extracts displayed the highest yield. In terms of antioxidant activity, the extract is found to have an inhibitory effect on lipid peroxidation. All manufactured extracts have biological properties, which opens up an extensive variety of potential claims in the nutrition and medicinal industries. Potentially, the extracts can be utilized as normal causes of antioxidants in place of synthetic substances for food preservation as well as the production of functional foods (Güllüce et al., 2003; Şahin et al., 2003).

The medicinal properties of summer savory are shown in Table 2.

**TABLE 2:** Medicinal uses of Summer Savory.

Disease type	Study design	Symptoms	Mechanism	Reference
Alzheimer’s	—	Lack of acetylcholine	Part of the *Satureja* spp. contains phenolic compounds such as flavonoids and flavonoid glycosides, which are sources of antioxidants able to diminish the development and evolution of Alzheimer’s disease and decrease neuronal degeneration	Öztürk (2012)
Inflammatory bowel disease	Mice	Inflammation	Phenolic acids help to reduce inflammation	Hajhashemi et al. (2002), Rocha et al. (2007)
			The polyphenols and essential oil of *Satureja* spp. exhibit significant anti-inflammatory activity. The literature reports the traditional employment of *S. hortensis* as a solution for inflammation diminishing and pain relief	
Cancer	Humans	Lump, abnormal bleeding, prolonged cough, unexplained weight loss, and a change in bowel movements	The effect of carvacrol on a human non-small cell lung cancer (NSCLC) cell line also named A549 demonstrated the inhibitory action of carvacrol on cancer cells. The research shows that carvacrol could present anti-carcinogenic activity and could be employed in cancer treatment	Koparal and Zeytinoğlu (2003), Kennedy et al. (2018)
Rhino-sinusitis	Rabbit	nasal discharge, sneezing, and swelling of the nose	The anti-inflammatory activity of *Satureja hortensis* L. was investigated by evaluating NO• metabolites. The results confirmed the potential of *Satureja hortensis* L. extract in inflammation reduction, thus suggesting its use in the treatment of rhino-sinusitis diseases	Uslu et al. (2003)
Heart-related	Humans	Difficulty in breathing, heartburn, nausea, vomiting, abdominal pain, and cold sweats	Literature states that extract of *S. hortensis* L. in methanol reduces blood platelet adhesion, aggregation, and secretion, thus explaining its traditional employment in cardiovascular and blood clot disease treatment	Mihajilov-Krstev et al. (2010)
Diabetes	Humans	Weight loss	Polyphenols are considered great natural antioxidants that have been demonstrated to act similarly to anti-diabetic medicines, which decrease the glucose concentration of blood	Ahmadvand et al. (2012), Ramachandran (2014)
		Frequent fatigue		
		Dry mouth		
		Burning and pain in feet		
		Itching		
		Decreased vision		
Hepatitis B	Humans		The antiviral properties of Savory spp. essential oil were investigated, and some results revealed that *Satureja boliviana* can slow down the actions of hepatitis B, herpes simplex type 1 virus (HSV-1), and vesicular stomatitis virus	Bezić et al. (2009)
				Momtaz and Abdollahi (2010)
Genotoxin	Rats	Oxidative stress	SHE (*S. hortensis* ethanolic extract) displayed a considerable inhibitory effect on oxidative DNA damage	Behravan et al. (2006)
			SHEO (*S. hortensis* essential oils) also displayed appreciable inhibitory activity on H_2_O_2_ induced chromosomal damage	
Antifungal	—	—	The essential oils extracted from plants have many advantages compared to traditional chemical fungicides, which makes their future use promising	Shirzad et al. (2011)
Antioxidant, Hepato-protective	Rats	Oxidative stress	A single dose of cisplatin (7.5 mg/kg) produced damage in the liver, as demonstrated by the rise in serum ALT, ALP, AST, and GGT contents. Adjuvant treatment with *S. hortensis* extract generated a considerable reduction in serum AST, ALT, and ALP quantities, demonstrating its hepatoprotective activity	Boroja et al. (2018)
Antinociceptive, Anti-inflammatory	Male mice	Inflammation	Decreased acetic acid-induced abdominal twitches. Hydroalcoholic extracts considerably lowered the pain responses in the early and late phases of the formalin test, while the polyphenolic extract and essential oil demonstrated effectiveness only in the late phase of the formalin test	Hajhashemi et al. (2012)
Antioxidant, Cytotoxic, Antibacterial	—	—	The extracts displayed antioxidant, cytotoxic, and antimicrobial activities, with the greatest biological potential exhibited in the case of subcritical water extracts	Mašković et al. (2017)
Antimutagenic	Humans	—	Phenylpropanoids and phenolic molecules like flavonoids, phenolic acids, and phenolic monoterpenes were proven to be responsible for antimutagenic activity in aromatic plants	Caillet et al. (2011)
Protective effect against AFB_1_ mutagen, Antioxidant	Humans	Increased MN frequencies, oxidative stress	Luteolin was demonstrated to possess many health benefits. Research regarding luteolin derivatives could contribute to the knowledge of their positive effects	Orhan et al. (2016)

**FIGURE 2:**
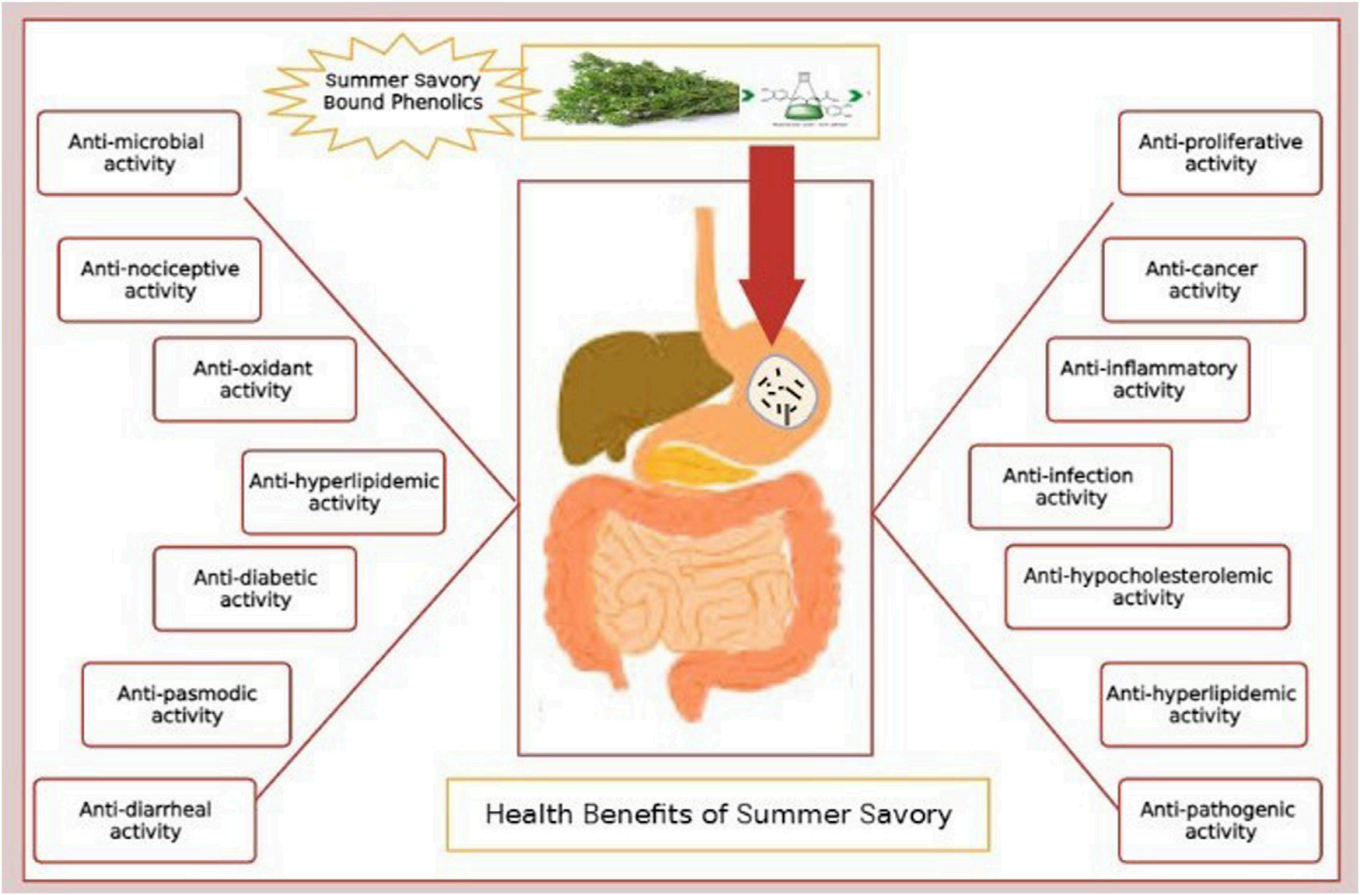
Prospective health benefits of summer savory.

Since most of the compounds found in the herb display specific biological properties (Figure 2), these are presented in the following subsections.

### 4.1 Antimicrobial activity of summer savory

There has been a slew of research in recent years focusing on the antibacterial properties of fragrant florae essential oils and their possible relevance in nutrition protection (Ghalfi et al.). Many studies have been published suggesting a relationship between the chemical construction of essential oil mechanisms and antibacterial properties. In particular, essential oils affect the cell membrane by interacting with and disrupting the phospholipid bilayer and by affecting enzyme activity and genetic resources in bacteria (Rezvanpanah et al., 2011). Essential oil of *S. hortensis* is rich in carvacrol and thymol, which are isomeric composites with a phenylic acid group in their structure. Both thymol and carvacrol suppress the diversity of microbes including bacteria and fungus (Adiguzel et al., 2007; Razzaghi-Abyaneh et al., 2008). The *Satureja* family contains phenolic compounds and their metabolites, which alter the permeability of the cell crust while inhibiting cell respiration. In this way, the *Satureja* family performs antibacterial activity. (Gursoy et al., 2009; Mahboubi and Kazempour, 2011). The antiseptic properties of various herbs and spices have long been known, and they have been utilized in food preservation and healing. (Omidbeygi et al., 2007). Apart from deterioration in foods, fungus development leads to undesired metabolites such as aflatoxin, which may be derived from Aspergillus species (A. parasiticus and A. flavus). Additionally, mycotoxin (toxigenic fungus) can be found in food and grains that have been preserved for a long time. In both animals and humans, aflatoxins are known to be strong hepatocarcinogens. To some extent, antifungal properties have been discovered in several savory species (Dikbas et al., 2008). Summer savory ingredients can be used as an additional preservative in food items due to their antibacterial properties (Rezvanpanah et al., 2011). The essential oil from some savory species has been shown to be high in antiviral properties (Bezić et al., 2009). The seeds and leaves of summer savory are good sources of essential oil. The essential oil of summer savory is composed of chemical compounds (hydrophobic and hydrophilic molecules) that play an important role in antimicrobial activity. The hydrophilic and hydrophobic molecules play a favorable role in antimicrobial activity. The outer layer of Gram-positive bacteria is a peptidoglycan cell wall that allows hydrophobic molecules to penetrate and reach the internal material, whereas the outer layer of Gram-negative bacteria is lipopolysaccharide, which allows mainly small hydrophilic molecules to pass and is only partly permissive for hydrophobic molecules. The hydrophobicity of essential oils is responsible for the disruption of microbial structures. The essential oil has different mechanisms of action on the microbial population, including degradation of the cell wall and cytoplasmic membrane, cytoplasm coagulation, and diffusion through the double lipid layer of the membrane, together with alteration of its permeability and function (Nazzaro et al., 2013). *In vitro* investigations have shown that the *Satureja boliviana* could suppress the overall effects of vesicular stomatitis virus (VSV), hepatitis B, and herpes simplex type 1 virus (HSV-1). However, S. montana can protect against HIV-1 virus (Omidbeygi et al., 2007). It has been discovered that *S. hortensis* essential oil has excellent antifungal action compared to *Aspergillus flavus.* Moreover, it could be exploited as a cause of environmental plant antifungals to protect various food products from infection and saprophytic fungi. *S. hortensis* essential oil has a broad antibacterial spectrum that inhibits the progress of the social and phytopathogenic bacteria, fungi, and yeasts that cause food spoiling. Vital oils from *S. hortensis* were found to be effective against *S. aureus, Listeria monocytogenes, S. typhimurium,* and *E. coli* O157:H7 along with the *Pseudomonas putida* strain obtained from meat (Oussalah et al., 2006). Essential oils from summer savory reduce the mycelial development of plant-pathogenic fungi (*Botrytis cinerea* and *Alternaria mali*) due to their antifungal properties (Boyraz and Özcan, 2006). Furthermore, the oil was shown to reverse the progress of aflatoxin (AFG1 and AFB1) by *Aspergillus parasiticus in vitro* under storage conditions (Dikbas et al., 2008), in liquid standard and in tomato adhesive (Omidbeygi et al., 2007), and reverse the development and production of aflatoxin (AFG1 and AFB1) by *A. parasiticus* (Razzaghi-Abyaneh et al., 2008). The antiseptic effect of *S. hortensis* essential oil was tested and compared to selected strains using the broth micro-well dilution method. Vital oil was found to be effective against entirely medical insulates from injuries that were verified. The oil has the best antibacterial action against *Acinetobacter spp*. and *S. aureus* (Pintore et al., 2002). It also displays activity against *Staphylococcus* spp. and E*. coli* (Wilkinson et al., 2003). In identical concentrations, the oil remains efficacious against *Enterobacter spp*. and *Enterococcus* spp.*, S. pyogenes, P. mirabilis*, (Wilkinson et al., 2003). The oil’s significant antibacterial activity is due to its high concentration of phenol component carvacrol, which has already been proven to have antimicrobial activity (Ben Arfa et al., 2006). Carvacrol has been found to have substantially strong antibacterial potential compared to other chemically similar compounds, such as eugenol (Ben Arfa et al., 2006).

### 4.2 Antioxidant activity of summer savory

Antioxidants are substances that prevent the oxidation of other compounds by preventing or delaying the beginning or proliferation of oxidative chain reactions (Roobab et al., 2018; Mukhtar et al., 2023). Fears about the antagonistic properties of artificial antioxidants have recently prompted the consumption of natural antioxidants found in all plants and all their parts (Bakkali et al., 2008). Numerous studies have proven that *Satureja* strains display antioxidant action (Exarchou et al., 2002). Previous studies have stated that the oils of *Satureja* species are a rich source of isopropanoids and flavonoids, such as p-cymene, linalool, carvacrol, thymol, β-caryophyllene, and γ-terpinene. These compounds have powerful antioxidant properties (Ruberto and Baratta, 2000). It has been determined that the antioxidant consequence of fragrant plants may be related to the existence of hydroxyl groups in their phenolic chemicals. In *S. montana,* components with hydroxyl groups have relatively significant antioxidant activity (Radonic and Milos, 2003). During storage, the *Satureja cilicica* essential oil showed substantial antioxidant activity in butter. Moreover, with increased concentrations of oil, the antioxidant properties of rose oil also increased (Ozkan et al., 2007). The outcomes of the study of Ozkan et al. (2007) show that the essential oil of *S. cilicica* can be employed as a natural antioxidant and fragrance agent in fat. Oxidative damage is induced by the formation of sensitive oxygen species (SOS) throughout ordinary cell aerophilic respiration, and it plays a key part in the start and progression of several illnesses in the human body. Antioxidants play a significant role in defending cells from oxidative compensations and in the prevention of a variety of diseases (Szőllősi and Varga, 2002). This study suggests that the methanolic extract of the S. hortensis aerial part may be valuable against cisplatin-induced oxidative damage in the liver, kidney, and testes of rats (Boroja et al., 2018). Another study verified that S. hortensis extract is a good source of antioxidants. However, different methods were used to measure antioxidant properties. The outcomes of FRAP, ABTS, and DPPH measured the high activity of the S. hortensis extract. Moreover, the total phenolic and total flavonoid content was also determined as high in S. hortensis extract (Mašković et al., 2017). Previous studies have proven that leaves and essential oil are important sources of chemical compounds including isopropanoids, rosmarinic acid, and flavonoids. The outcomes confirm that both leaves and oils have a high antioxidant capacity (Momtaz and Abdollahi, 2010; Chkhikvishvili et al., 2013).

#### 4.2.1 Summer savory protects Jurkat T Cells against oxidative stress

**FIGURE 3:**
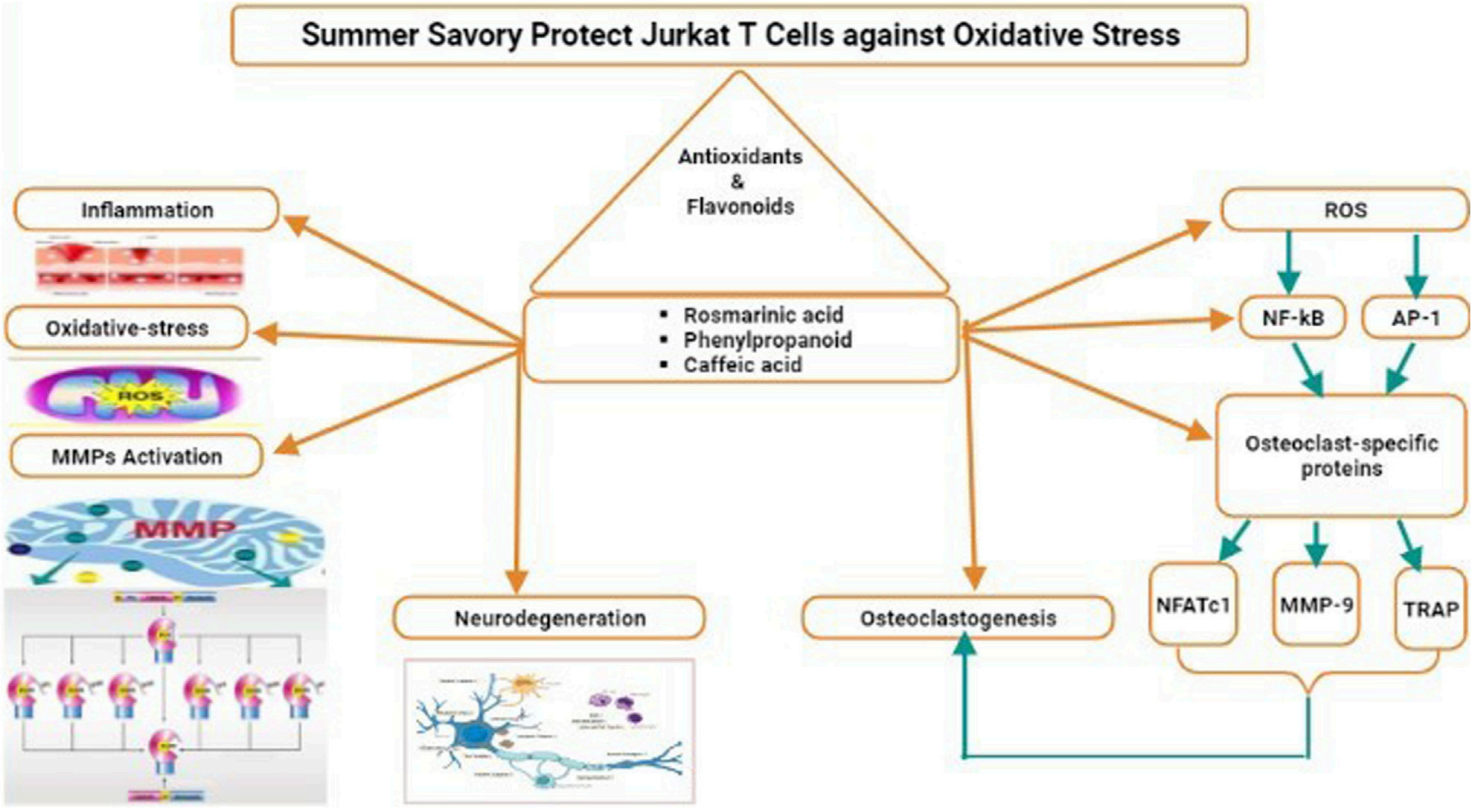
Potential role of summer savory in Jurkat T cell against oxidative stress.

Rosmarinic acid is the main phenylpropanoid component in summer savory. Jurkat cells can be protected against oxidative stress generated by hydrogen peroxide by *S. hortensis* and its rich rosmarinic acid proportions. The results of Hajhashemi et al. (2002), Sanbongi et al. (2003), Osakabe et al. (2004) remain consistent through the cytoprotective, antiinflammatory, and antioxidant activities of *S. hortensis* (Hajhashemi et al., 2002) and rosmarinic acid (Sanbongi et al., 2003; Osakabe et al., 2004) in animals and humans. *S. hortensis* extracts exhibited significant protective antioxidant actions when administered to H_2_O_2_-stressed lymphocytes isolated from healthy rats’ blood (Behravan et al., 2006). Rosmarinic acid protected human neuronic cells from hydrogen peroxide-induced apoptosis in cell cultures (Lee et al., 2008) and inhibited the creation of reactive nitrogen and oxygen species in RAW264.7 macrophages encouraged with phorbol 12-myristate 13-acetate or lipopolysaccharides in a dose-dependent manner. However, maximum phenolic composites demonstrated pro-oxidant properties at small dosages in a metal catalyst system before switching to antioxidant activity at higher concentrations (Fukumoto and Mazza, 2000). Furthermore, high dosages (2–3-mM) of caffeic acid and phenylpropanoids have recently been demonstrated to protect Jurkat cells from H_2_O_2_-induced DNA impairment by chelating intracellular labile iron (Kitsati et al., 2012). In addition to rosmarinic acid, the existence of powerful antioxidants in the phenolic element may enhance its antioxidant potential. *S. hortensis* may also aid in the neutralization of hydrogen peroxide by increasing the activity of antioxidant enzymes. In Jurkat cells, SOD and Catalase play crucial roles in the regulation of apoptosis and oxidative stress (Kagan et al., 2002). The extract of *Perilla frutescens* rosmarinic acid in water from a Lamiaceae plant was demonstrated to have effects on the protein and mRNA appearance of the antioxidant enzymes in cultivated human vein endothelial cells (Saita et al., 2012). Anti-inflammatory features like IL-10 may be generated in stressful settings to counteract the rapid rise in pro-inflammatory cytokines and regulate the amount and interval of the inflammatory response. The accumulation of antioxidant-rich herbal ingredients in the diet of wildlife suffering from pro-inflammatory conditions has been demonstrated to enhance IL-10 levels (Kim et al., 2011) or both IL-2 and IL-10 (Zhang et al., 2012) in tandem, with a decrease in pro-inflammatory markers like IL-6, TNF-α, and IL-1β levels. Furthermore, nutritional interferences conserved standard antioxidant enzyme action, prevented fat peroxidation, and boosted HDL levels in the preserved animals, leading to improved immunity and the alleviation of diseases (Zhang et al., 2012). In a lipopolysaccharide-stimulated macrophage model, rosmarinic acid boosted 1L-10 productions (Mueller et al., 2010). Adding the *S. hortensis* extract or its phenolic component to H_2_O_2_-challenged Jurkat cells restored survival and proliferation, relieved the G0/G1 arrest, and regulated apoptosis. Overall, these findings are consistent with the overall reaction of cells to oxidative stress, whereby small quantities of reactive oxygen species encourage cell growth, in-between measures cause evolution arrest, and high oxidative stress causes cell death through necrotic or apoptotic mechanisms (Martindale and Holbrook, 2002). The accumulation of *S. hortensis* extracts appears to reduce the oxidative stress caused by hydrogen peroxide on the cells. These effects may be related to phenolic compounds and rosmarinic acid direct radical-scavenging activity but also to unintended processes like the increase in antioxidant enzymes and the production of anti-inflammatory gesturing particles like IL-10 (Figure 3).

#### 4.2.2 Fe (III) reductive and free radical-scavenging properties of summer savory

The capacity of complexes and herb extracts to decrease Fe (III) is frequently employed as a measure of electron contributing procedure. This may be due to the phenolic antioxidant mechanism (Yildirim et al., 2000). The components of Summer Savory are resolvable in acidic aqueous methanol solvent and they have the ability to donate electrons to the unbalanced free radicals (Dorman et al., 2003). These are further constant non-reactive classes, with the EtOAc-soluble mechanisms acting as maximum active electron donors (Dorman et al., 2003). ABTS and DPPH artificial free radicals and hydroxyl radicals are used to examine the possible free radical-scavenging actions of the *Satureja hortensis* L. extract and subfractions (Jayasinghe et al., 2003). ABTS and DPPH are widely employed to estimate free radical-scavenging capabilities (Jayasinghe et al., 2003). There are often problems with solubility and interference with DPPH tests; thus, ABTS free radicals are frequently utilized (Arnao, 2000). However, it has been suggested that these approaches are not able to use a biologically or food-related reactive species and oxidizable substrate. Moreover, they can only indicate potential antioxidant activity and, therefore, provide no direct information on defensive presentation (Güllüce et al., 2003). Phospholipids are thought to play a key role in off-flavor development and oxidative deterioration in foodstuffs (Frankel and Meyer, 2000). The capability of the models to prevent ascorbate-Fe (III)-generated hydroxyl radical-mediated peroxidation of a heterogenous phospholipid-aqueous phosphate buffered system is resolute, and the hydroxyl radical is an extremely volatile radical that is used in in vivo study. *In vitro* studies have shown that the EtOAc-soluble mechanisms are activated at the maximum level, including crude extract and Fe (III) decrease assay. With hexane, EtOAc, and n-BuOH, an aliquot part of this extract was sub-fractioned by liquid-liquid breakdown against water (Kim et al., 2003). Fe (III) reduction assays as well as ABTS, DPPH, and hydroxyl free radical-scavenging assays were used to characterize these materials’ antioxidant capabilities (Hajhashemi et al., 2000). The EtOAc fraction and crude extract were the maximum active samples, showing much lower activity (Antolovich et al., 2002). The crude extract containing EtOAc-soluble components had predominantly significant action when used as preservative to prevent free radical-mediated destruction of vulnerable components. Moreover, free radicals play a key role in the deterioration of human health. The EtOAc subfraction may have favorable impacts on human biology when it is used in adequate amounts in foods (Niki et al.).

### 4.3 Alzheimer’s disease

Alzheimer’s disease is generally caused by a deficiency of acetylcholine, which is a neurotransmitter. Alzheimer’s disease has been treated using acetylcholinesterase inhibitor tablets. Nevertheless, these treatments may involve adverse side effects. To prevent this sort of disease, the production of natural chemicals with antioxidant and anticholinesterase properties is preferable (Öztürk, 2012). Antioxidants can play a significant role as neuroprotective agents at the initial stage of Alzheimer’s disease (Silva et al., 2009). Some *Satureja* species are vital sources of phenolic compounds (flavonoids and flavonoid glycosides) that can play an important role as antioxidants. However, the antioxidant potential of *Satureja* species may reduce the chances of developing Alzheimer’s disease and neuronal degeneration (Öztürk, 2012). The activation of cell signaling pathways occurs due to oxidative stress. The results of Pritam et al. (2022) show reduced formations of toxic substances that foster the development of the disease. Antioxidants reduce free-radical-mediated damage in neuronal cells through detoxification. Moreover, the balance between antioxidants/oxidants is unfavorably unbalanced, which can have detrimental effects, such as Alzheimer’s disease (Sinyor et al., 2020). One study shows that the phenolic content of several *Satureja* species, specifically flavonoids and flavonoid glycosides, which delay the growth of Alzheimer’s disease and lower neural degeneration due to potent antioxidants (Öztürk, 2012). Thymol and carvacrol are abundant in *Satureja* species that can work as low cholinesterase inhibitors and protect people from oxidative stress and amnesia despite causing no adverse side effects (Öztürk, 2012). The study of Ross et al. (1999) shows that the progression of Alzheimer’s disease can be reduced by reducing neuronal damage. Previous studies have verified that different savory species can protect against various chronic diseases, including Alzheimer’s, diabetes, cancer, and cardiovascular diseases (Hamidpour et al., 2014).

### 4.4 Cancer

The plant materials are composed of phenolic chemicals that can help to protect or reduce oxidative destruction by both non-free-radical and free-radical species (Alizadeh et al., 2010; Ali et al., 2022). Regulation of oxidative chain reaction formation and growth helps to prevent various disorders, including oxidative stress dysfunctions, cancer, heart disease, and neural disorders (Szőllősi and Varga, 2002; Alizadeh et al., 2010). The anti-carcinogenic, vasoprotective, anti-allergic, anti-proliferative, anti-inflammatory, and antimicrobial properties of phenolic acids and flavonoids have been documented in several studies. *Satureja montana *L. has been used as medication in the treatment of several types of cancer (Cetojevic-Simin et al., 2008). The extracts suppressed the development of HT-29 (human colon adenocarcinoma) cells at levels over 0.7 mg/mL, and HeLa (human cervix epidermoid carcinoma) was shown to present the maximum sensitivity to the savory extract. Several *Satureja* spp. species are powerful sources of antioxidants that can prevent the growth of an extensive variety of human tumor cells (Cetojevic-Simin et al., 2008). Food products have been demonstrated to be effective and stable sources of innovative medicine. Carvacrol is a monoterpene present in the essential oils of a diversity of fragrant plants. The activity of carvacrol on the human non-small cell lung cancer (NSCLC) cell line named A549 indicates that it reduces cancer cells but has no impact on normal lung cells (HFL1). The findings of Koparal and Zeytinoğlu (2003) suggest that carvacrol has anti-carcinogenic properties and can be used as a cancer treatment medicine.

### 4.5 Anti-infection properties

Bacteria, fungi, and viruses are the most common sources of illnesses that affect both flora and fauna. Plant essential oils act as secondary metabolites to protect humans from natural enemies. It may be genetic or in reaction to pathogens. The antimicrobial activity of *Satureja* spp. was initially discovered in the 1950s, and it was discovered that the inhibitory action of savory is probably due to its high carvacrol and thymol content. These are the two most effective herbal antiseptic compounds (Oussalah et al., 2006). Essential oil concentration and composition are influenced by storage circumstances as well as the concentration and type of the mark microorganism (Baydar et al., 2004). Essential oil concentrations in *Satureja parnassica* and *Satureja thymbra* have been found to fluctuate. The oils extracted during the flowering time were have been determined as the most potent, with minimum inhibitory concentration (MIC) standards and significant antibacterial activities (Chorianopoulos et al., 2006). Essential oils have been shown to have inhibitory effects against a diverse variety of food-deteriorating bacteria due to their concentration in valuable components (Skočibušić et al., 2006). Furthermore, different varieties of *Satureja* have also been widely studied for their resistance to foodborne diseases. In Greece, the essential oils extracted from *Satureja* spp. contain monoterpene hydrocarbons and phenolic monoterpene. These oils have outstanding antibacterial properties against foodborne pathogens (Chorianopoulos et al., 2004). Summer savory (*S. hortensis* L.) extract was investigated in relation to the mycelial growth of food fungi due to the fungicidal activity of the hydrosol (Boyraz and Özcan, 2006). The extract was found to have dose-dependent fungicidal activity at all dosages. Carvacrol has been shown to be most effective in *S. thymbra*, followed by p-cymene and monoterpene hydrocarbons c-terpinene (Soković et al., 2002). The effect of *S. boliviana* against binary distinct VSV and viruses-HSV-1 has also been investigated. The active component in the aqueous extract of *S. montana* has been discovered to be non-polar water-soluble molecules. Non-polar chemicals like essential oils and extracts have substantial anti-HIV-1 action (Abad et al., 1999).

### 4.6 Cardiovascular diseases

Cardiovascular issues and thrombosis (blood accumulations in the vein or artery) occur due to platelet hyperactivity that contribute to their adsorption to the vascular wall (Yazdanparast and Shahriyary, 2008). Cardiovascular diseases (CVD) are usually illnesses that are directly related to blood arteries and the heart. Excessive oxidative stress produces reactive oxygen species (ROS) that are responsible for the pathophysiology of different CVDs such as ventricular remodeling, cardiomyopathy, myocardial infarction, heart failure, cardiac hypertrophy, and atherosclerosis. The body’s endogenous system fails to maintain normal physiology due to excessive oxidative stress. However, different sources of antioxidants are necessary to scavenge free radicals (Jain and K Mehra N, 2015). Different investigations have shown that *S. hortensis* has anticoagulant blood properties. Flavonoid, monoterpene hydrocarbons, and carvacrol, including phenolic acids and apigenin (labiatic acid), may all play a role in *S. hortensis* anti-platelet activity (Yazdanparast and Shahriyary, 2008). The methanol extract of *S. hortensis* has been shown to reduce secretion, aggregation, and blood platelet adhesion, which may explain its conventional usage in the treatment of blood clots and cardiovascular issues (Mihajilov-Krstev et al., 2010). The current study of Khalid et al. (2023) suggests that essential oil, antioxidants, and phenolic compounds protect CVDs by reducing cholesterol, preventing oxidation, and reducing platelet aggregation, respectively. Previous studies have suggested that phenolic compounds reduce the risk of CVDs by preventing atherothrombosis and platelet activity (Torres-Urrutia et al., 2011; Fuentes et al., 2012).

### 4.7 Antidiabetic and anticholesterol effects

Hypertension and hyperlipidemic raise the threat of cardiac ailment. It has been recognized that hypercholesterolemia has been linked to a variety of clinical conditions including atherosclerosis, diabetes mellitus, thromboembolic, and cardiovascular diseases. Antioxidants play a vital role in the handling of illnesses involving oxidative stress damage (diabetes mellitus) (Vosough-Ghanbari et al., 2010). Antioxidant medication is the best way to prevent and decrease the development of diabetic consequences, including hyperlipidemia and liver issues. Natural antioxidants derived from medicinal herbs have recently captivated researchers’ curiosity as a possible replacement for artificial antioxidants (Ahmadvand et al., 2012). Polyphenols are well-recognized natural antioxidants that have been shown to reduce blood glucose levels in a manner comparable to antidiabetic medications. *Satureja khuzestanica* (SKE) is an Iranian *Satureja* plant with antioxidant activities and anti-diabetic actions (Momtaz and Abdollahi, 2010). Malondialdehyde levels and serum glucose were controlled in diabetic patients using SKE (Momtaz and Abdollahi, 2010). SKE essential oil has been investigated for its hepatoprotective, hypolipidemic, and antiatherogenic properties. In diabetic patients, it can reduce the risk of cardiovascular mortality and liver injury (Ahmadvand et al., 2012). Thymol and carvacrol are the essential components of *Satureja* species. Essential oils have been revealed to reduce serum cholesterol levels. Flavonoids are abundant in *Satureja* species that have been shown to have anti-hyperlipidemic and antioxidant effects. SKE has been shown to significantly reduce the triglyceride levels and fasting blood glucose in hyperlipidemic and diabetic rats along with ATP levels and lipid peroxidation in numerous trials (Momtaz and Abdollahi, 2010). The study of Momtaz and Abdollahi (2010) indicates that some *Satureja* species, including SKE, can be utilized as an enhancement in hyperlipidemic diabetic patients due to their lipid-lowering and antioxidant characteristics (Vosough-Ghanbari et al., 2010). The flavonoids extracted from summer savory reduce cholesterol in rabbits and lead to a considerable decrease in serum cholesterol (Mchedlishvili et al., 2005). Rats ingesting *S. khuzestanica* essential oils showed a considerable increase in total antioxidant capacity and a reduction in normal blood lipid peroxidation levels *S. khuzestanica* oil’s antioxidant properties may explain its triglyceride-lowering properties (Abdollahi et al., 2003). Isopropanoids such as carvacrol, thymol, and flavonoids are also important components of *S. khuzestanica*. Thymol and carvacrol have been demonstrated to lower serum cholesterol stages considerably. Flavonoids have also been revealed to have anti-hyperlipidemic effects (Momtaz and Abdollahi, 2008).

### 4.8 Anti-inflammatory and analgesic effects

Inflammation is the body’s natural defense process in relation to pathophysiological conditions. Several examinations have been conducted in order to discover extra influential anti-inflammatory treatments with less harmful properties (Amanlou et al., 2005). Plants of the Lamiaceae family are recognized for their pain-relieving and antispasmodic effects. In animal experiments, many components of *Satureja* species (flavonoids) have been found to be essential for analgesic, relaxing, and vasodilatory actions (Momtaz and Abdollahi, 2010). In addition to analgesic effects, various *Satureja* species have been identified to exhibit anti-inflammatory properties (Momtaz and Abdollahi, 2010). *S. hortensis* L. has been used as a bone pain and muscular reliever in traditional remedies Previous studies have shown that polyphenolic and essential oil from *Satureja* spp. have strong anti-inflammatory characteristics. The research confirms the traditional use of *S. hortensis* as a pain killer and an anti-inflammatory (Hajhashemi et al., 2002). According to several investigations, *Satureja* species, including *S. hortensis* and SKE, act as anti-inflammatory medications and are equivalent to morphine, indomethacin, and prednisolone (Momtaz and Abdollahi, 2010). Animal and human trials have shown the anti-inflammatory and anti-allergic actions of savory linked to the polyphenolic fraction (rosmarinic acid) (Chkhikvishvili et al., 2013).

### 4.9 Summer savory and reproduction stimulatory effects

A study was conducted on male rat fertility in which *S. khuzestanica* essential oil significant gains in fertility index, fecundity, litter index, and potency Additionally, it reduced the post-implant loss (Haeri et al., 2006). Moreover, the seminal vesicles, ventral prostate, and weights of the testes were greatly elevated, and the weight of the testes, testosterone, and FSH were concentrated. These alterations could be linked to the antioxidant potential of the essential oils. The principal antioxidants in *Satureja* spp. are pcymene, carvacrol, and flavonoids. The results indicate the stimulatory effects of this genus on reproduction (Radonic and Milos, 2003).

## 5 Applications of summer savory

**FIGURE 4:**
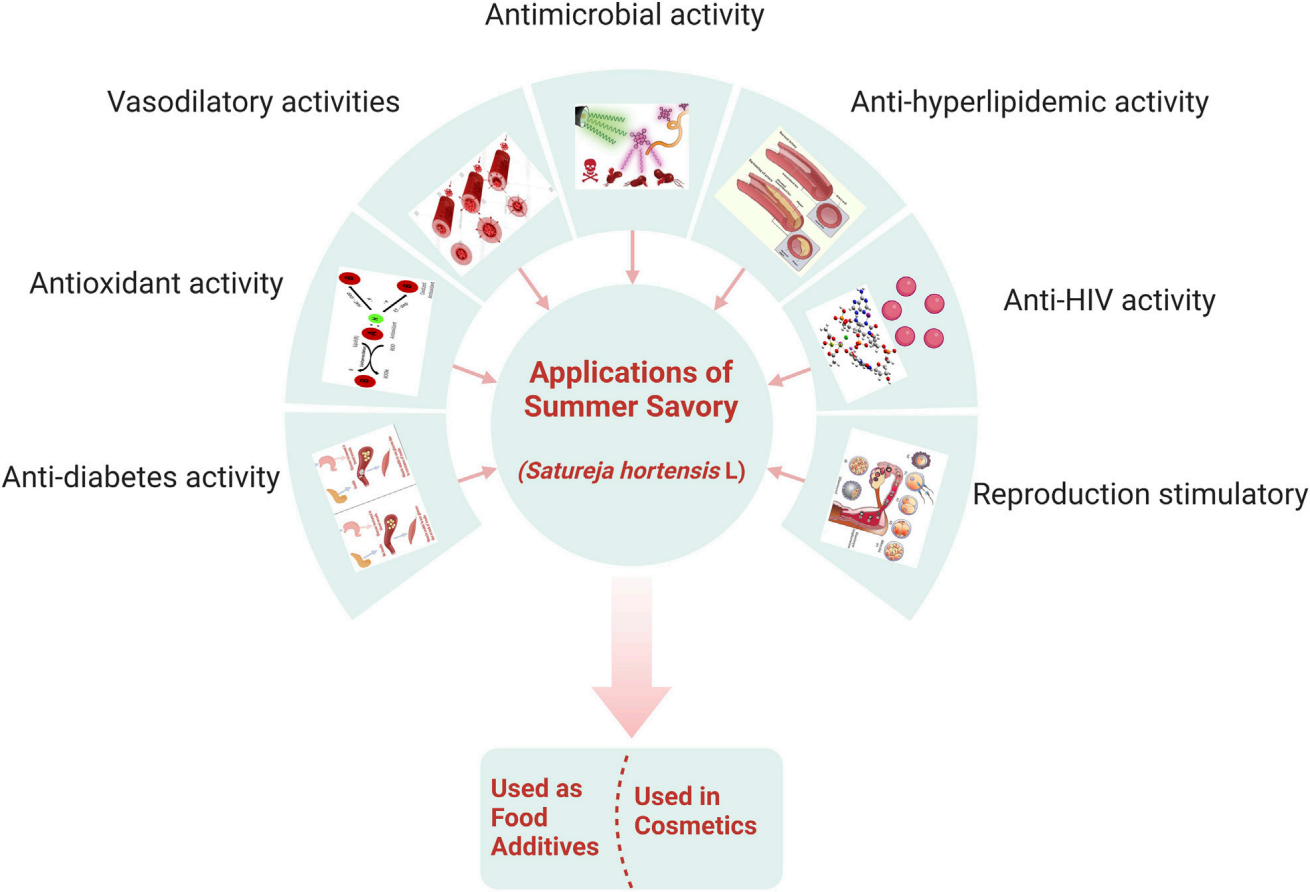
Different applications of summer savory.

*S. hortensis* is used worldwide as a food additive, flavoring, and spice and in herbal beverages due to its pre-eminent ethnomedical activity and pharmaceutical and food applications (Figure 4). Furthermore, summer savory oil has been used in the cosmetic industry and in perfumes, both alone and in combination with essential oils (Sefidkon et al., 2004).

Primarily, savory species are used to treat muscle pain, flatus, and intestinal disorders such as indigestion, cramps, nausea, and diarrhea (Abdollahi et al., 2003). Other properties of *S. hortensis* include antifungal, antibacterial, antioxidant, anti-hyperlipidemic, anti-HIV, anti-diabetes, expectorant, reproduction stimulatory, and vasodilatory activities (Şahin et al., 2003; Amanlou et al., 2005; Basiri et al., 2007). In ancient medical books, it is demonstrated that *Satureja* spp. has a medicinal impact on respiratory diseases, such as coughs and asthma (Vagionas et al., 2007).

### 5.1 Summer savory used as a native additive in broiler feed

**FIGURE 5:**
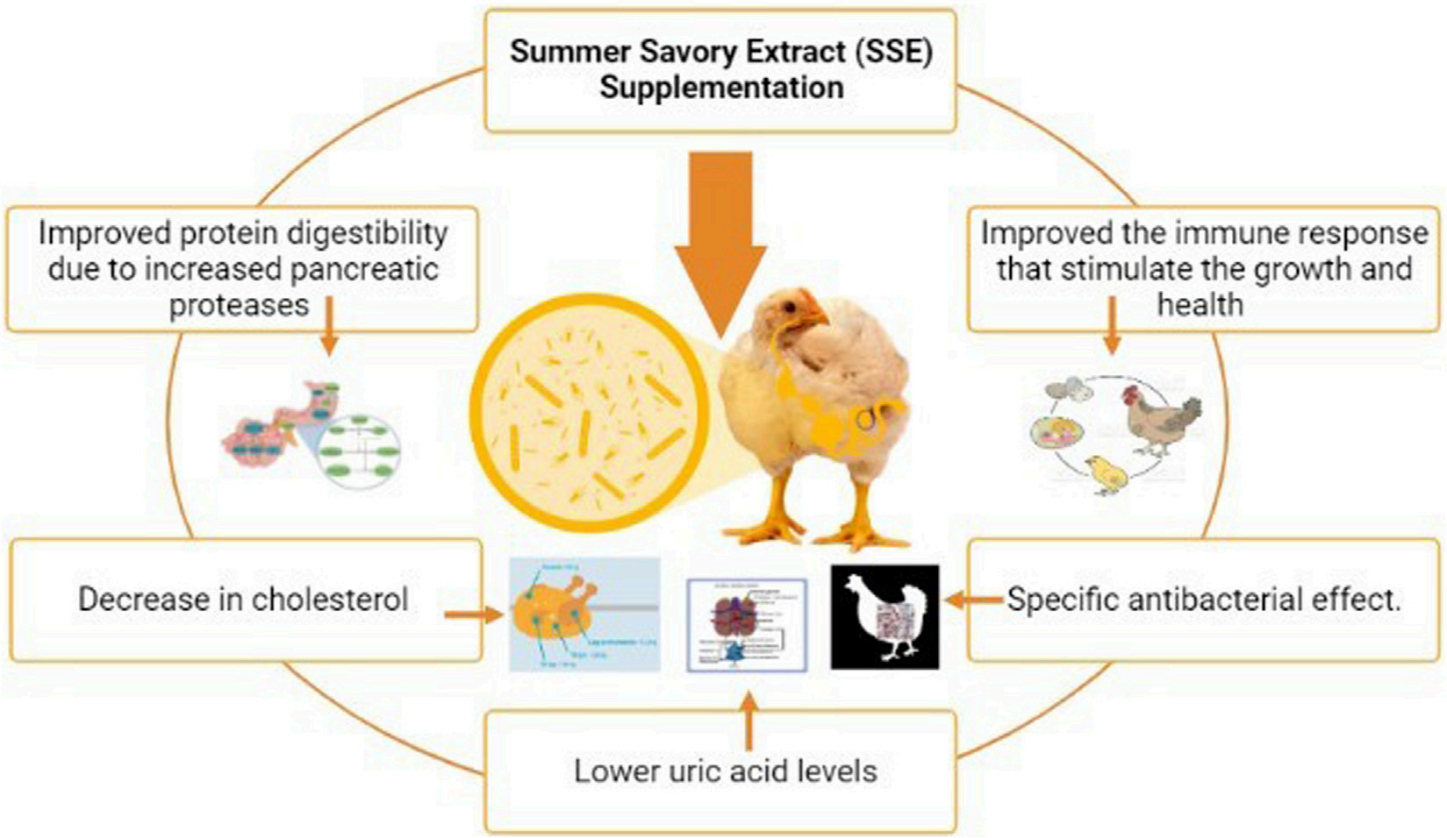
Summer savory use as supplementation.

Essential oils have been revealed to have various favorable properties in broiler feed, including increased feed intake (Jang et al., 2007), improved digestibility, higher digestive enzyme secretion (Jamroz et al., 2005), and microbial ecosystem balancing (Liolios et al., 2009). The beneficial qualities of the essential oils of this plant may account for the increased performance reported, with the addition of summer savory essence (SSE). The plasma content of feed could be linked to the effects of the essential oils in summer savory foods on digestion. In broilers, feed supplementation with thymol was found to greatly improve pancreatic action (Lee et al., 2003). Protein digestibility improved due to increased pancreatic proteases, which could explain why the SSE-supplemented sets had lower uric acid levels. The cholesterolemic action of essential oils (Nobakht et al., 2012) could explain the considerable decrease in cholesterol SSE addition in the diet at 200 and 300 ppm, along with LDL at 300 and 400 mg/kg SSE. SSE-supplemented diets improved the immune response of broilers as they grew older; the immune response, being dependent on age, may be attributable to the essential oil content and antioxidants (Attia and Al-Harthi, 2015). The considerable decrease in *E. coli*, correspondence to *Lactobacillus spp*., and a greater ratio of *Lactobacillus* to coliform suggest that SSE-treated birds have better gut health. This array of IB and IBD virus titers, *Lactobacilli* counts, and *E. coli* may indicate that savory extract has a specific antibacterial effect (Attia et al., 2017). Furthermore, better gut biology could explain the rise in antibody titers due to the nutrition sparing-effect (Attia et al., 2017). Although there was no substantial impact of SSE supplementation in broiler feed, conversion ratio and body weight gain were considerably enhanced when 400 mg/kg SSE was used (Bombik et al., 2012). SSE supplementation enhanced the majority of the blood indicators and immunological response criteria evaluated (Bombik et al., 2012). *Lactobacilli* count was unaffected by diet. However, SSE lowered the *Escherichia coli* count and improved the *Lactobacillus* to coliform ratio (Bombik et al., 2012). Supplementing the broiler feed up to 400 mg/kg with SSE maintained growth features and increased health and feed efficiency (Pourhossein et al., 2015). There was no impact on broilers’ weight gain or feed intake during the initial growth period (Hajhashemi et al., 2002; Sanbongi et al., 2003; Uslu et al., 2003; Osakabe et al., 2004; Nybe, 2007; Can Baser, 2008; Brown, 2009; Burlando et al., 2010; Cutler et al., 2010; Rodov et al., 2010; Tavafi and Ahmadvand, 2011; Akhalkatsi et al., 2012; Gelovani et al., 2012; Hashemipour et al., 2014) (Ghazvinian et al., 2018). During the 15–28-day growth phase, broilers were fed up to 300 mg/kg SSE, and the feed intake was gradually lowered (Ghazvinian et al., 2018). During the finisher phase (Adiguzel et al., 2007; Brighente et al., 2007; Kizil et al., 2009; Mahboubi and Kazempour, 2011; Hamidpour et al., 2014; Aadil et al., 2015; Cvetanović et al., 2017; Katar et al., 2017; Mašković et al., 2017; Mohtashami et al., 2018; Maqbool et al., 2022; Arshad et al., 2023; Fatima et al., 2023; Waliat et al., 2023), dietary interventions had no effect on any of the growth indices examined. Finally, dietary supplementation with summer savory extract at 400 mg/kg as a natural feed addition helped broiler chickens maintain their growth and enhance their health (Daferera et al., 2000). (Figure 5).

## 6 Summer savory and toxicity

Toxicity is a condition in which chemical compounds or parts of a chemical mixture can damage an organ. Antioxidants are consumed on a large scale as nutraceuticals and food supplements that can preserve optimal health. It is well known that “excess of everything is bad”, yet it is not generally recognized that a high intake of antioxidants may also have adverse effects. On the other hand, some antioxidants are used to illustrate general considerations on the toxicity of antioxidants. The toxicity of antioxidants can be evaluated with some recommendations, including classical safety factors, the knowledge of the efficacity mechanism, bio-kinetic/bio-efficacy modeling, and antioxidant supplementation changes into therapy being also of interest (Bast and Haenen, 2002). The study of Boroja et al. (2018) suggests that the methanolic extract of the summer savory aerial part protects against cisplatin-induced oxidative damage. Cisplatin was induced to produce toxicity using intraperitoneal injection (Boroja et al., 2018). The results confirm that summer savory could be a valuable source of dietary and therapeutic phenolic compounds. However, summer savory can maintain normal health conditions or may be a remedy for different oxidative damage diseases (Boroja et al., 2018). Furthermore, there are fewer known side effects of *S. hortensis*, but people who are taking medication for chronic diseases are cautioned about its use. The advice of a healthcare provider is mandatory before starting any new therapy or consumption of medicinal plants. *S. hortensis* is not recommended for children, pregnant women, or breastfeeding women due to a lack of sufficient evidence of its safe use in these populations (Hamidpour et al., 2014). In general, essential oils are comprised of a large variety of elements; hence, they do not appear to have any unique cellular targets. The presence of phenols, aldehydes, and alcohols in essential oils contributes to their cytotoxic effect (Bakkali et al., 2008). It appears that a distinction can be made between *Satureja* spp. and poisonous effects in eukaryotic cells and cytotoxic effects on microorganisms (yeast, viruses, fungi, and bacteria). The anti-pathogenic action of *Satureja* spp. has been well documented. Many people throughout the world consume *Satureja* spp. in the form of spice, herbal tea, and extracts. *S. hortensis* is consumed as a vegetable on a daily basis and has no known negative effects (Hajhashemi et al., 2000). Furthermore, an *in vitro* study showed that both the ethanolic extract and the essential oil of summer savory protected rat lymphocytes from hydrogen peroxide-induced damage (Behravan et al., 2006). In rats, the essential oil of *S. khuzestanica* was found to protect against the toxicity of Malathion (a commonly used organophosphorus) (Basiri et al., 2007). The leaves of *S. gilliesii* contain two isomeric monoterpene peroxides and they were found to be poisonous to *Artemia salina* in other investigations (brine shrimp bioassay) (Labbe et al., 1993).

## 7 Conclusion

It is concluded that the leaves and seeds of summer savory (*Satureja hortensis* L.) contain different chemical components. Summer savory leaves are abundant in total phenolic components, especially flavonoids and rosmarinic acid. It has strong antioxidant, antimicrobial, and antifungal effects that play a preventative role in human health. Furthermore, their oxidant activity suppresses the growth of many large tumor cells and the growth of HT-29 (human colon adenocarcinoma) cells. Carvacrol and Thymol are suppressing a rich diversity of microbes in *S. hortensis*, which have medicinal properties such as anti-diabetic, antispasmodic, anti-hyperlipidemic, anti-inflammatory, anti-proliferative, and anti-nociceptive properties.
